# Extracellular Zinc Competitively Inhibits Manganese Uptake and Compromises Oxidative Stress Management in *Streptococcus pneumoniae*

**DOI:** 10.1371/journal.pone.0089427

**Published:** 2014-02-18

**Authors:** Bart A. Eijkelkamp, Jacqueline R. Morey, Miranda P. Ween, Cheryl-lynn Y. Ong, Alastair G. McEwan, James C. Paton, Christopher A. McDevitt

**Affiliations:** 1 Research Centre for Infectious Diseases, School of Molecular and Biomedical Science, University of Adelaide, South Australia, Australia; 2 School of Chemistry and Molecular Biosciences, Australian Infectious Diseases Research Centre and Institute for Molecular Bioscience, University of Queensland, Brisbane, Australia; CINVESTAV-IPN, Mexico

## Abstract

*Streptococcus pneumoniae* requires manganese for colonization of the human host, but the underlying molecular basis for this requirement has not been elucidated. Recently, it was shown that zinc could compromise manganese uptake and that zinc levels increased during infection by *S. pneumoniae* in all the niches that it colonized. Here we show, by quantitative means, that extracellular zinc acts in a dose dependent manner to competitively inhibit manganese uptake by *S. pneumoniae*, with an EC_50_ of 30.2 µM for zinc in cation-defined media. By exploiting the ability to directly manipulate *S. pneumoniae* accumulation of manganese, we analyzed the connection between manganese and superoxide dismutase (SodA), a primary source of protection for *S. pneumoniae* against oxidative stress. We show that manganese starvation led to a decrease in *sodA* transcription indicating that expression of *sodA* was regulated through an unknown manganese responsive pathway. Intriguingly, examination of recombinant SodA revealed that the enzyme was potentially a cambialistic superoxide dismutase with an iron/manganese cofactor. SodA was also shown to provide the majority of protection against oxidative stress as a *S. pneumoniae* Δ*sodA* mutant strain was found to be hypersensitive to oxidative stress, despite having wild-type manganese levels, indicating that the metal ion alone was not sufficiently protective. Collectively, these results provide a quantitative assessment of the competitive effect of zinc upon manganese uptake and provide a molecular basis for how extracellular zinc exerts a ‘toxic’ effect on bacterial pathogens, such as *S. pneumoniae*.

## Introduction

Metal ions are essential for all known forms of life. In a biological context, nearly one-third of all proteins require interaction with a metal cofactor to facilitate their activity [1]. The *d*-block ion manganese, which exists as the divalent cation Mn(II), is an essential trace element for almost all organisms and serves in a wide range of enzymes involved in phosphorylation, hydrolysis, carbon metabolism, decarboxylation, and oxidative stress response [2]. Manganese also has a particularly prominent role in protection against oxidative stress and is often a cofactor in superoxide dismutase (SOD), where it catalyzes the one-electron dismutation of superoxide to oxygen and hydrogen peroxide. The crucial role of Mn(II) in cellular function has been highlighted by its importance in the virulence of a number of bacteria, including *Bacillus anthracis, Staphylococcus aureus, Streptococcus (S.) pneumoniae,* and *S. pyogenes* [3]–[6]. *S. pneumoniae* (also known as the pneumococcus) is one of the world’s foremost bacterial pathogens [7]. Although this Gram-positive bacterium is typically carried asymptomatically in the human nasopharynx in a large proportion of the population, it is capable of spreading to the lungs and other tissues where it causes a range of serious diseases including pneumonia, meningitis, otitis media, and bacteraemia [7], [8]_ENREF_2. The greatest burden of pneumococcal disease occurs in developing countries. Pneumonia, of which *S. pneumoniae* is the most common causal agent, accounts for more than 1 million deaths per year, primarily in children under 5 years of age from developing countries [9]–[11]. In developed countries, despite the availability of vaccination and antimicrobial therapies, pneumococcal morbidity and mortality remains substantial [8], [12]–[14]. Deaths from pneumococcal disease predominantly occur in individuals over 60 years of age, with fatality rates of up to 20% for pneumonia and up to 60% for bacteraemia [7]. Essential to the capacity of the pneumococcus to mediate virulence is its ability to adapt and colonize different host niches.

*S*. *pneumoniae* is an aerotolerant anaerobe that, although it lacks catalase [15], is capable of detoxifying reactive oxygen species (ROS) and peroxide by use of SOD and a thiol-peroxidase (PsaD). The concerted actions of these enzymes enable the pneumococcus to not only tolerate molecular oxygen, but to safely consume it via reduction by cytoplasmic NADH oxidase (Nox) and pyruvate oxidase (SpxB) [16]–[18], thereby increasing the yield of ATP per mole of glucose. Protection against oxidative stress is intimately linked to cellular abundance of two transition row metals, iron and manganese. By contrast with many other pathogens, the pneumococcus has a relatively low requirement for iron. This is presumably due to the lack of a complete respiratory chain and the presence of only a few iron-sulfur containing proteins [19]–[21]. As a consequence, the lower abundance of iron in the pneumococcus reduces the possibility that hydroxyl radicals (^.^OH^−^) could arise from the Fenton reaction of ferrous ions [Fe(II)] and hydrogen peroxide (H_2_O_2_).

Manganese is specifically acquired by *S. pneumoniae* via the cell-surface lipoprotein PsaA, and this recruitment is essential for pneumococcal colonization and virulence in the human host [4], [22], [23]. PsaA belongs to the Cluster A-I subgroup of solute-binding proteins (SBPs) and interacts with an ATP-binding cassette (ABC) transporter, PsaBC, to deliver the recruited ion into the cell. Metal ion ABC permeases, which employ these types of SBPs, do not have discrete binding sites for the ion and, as such, all cargo specificity derives from the SBP [24], [25]. Recently, work from our group identified that despite the physiological role of PsaA in Mn(II) acquisition, the protein was capable of binding either its cognate ligand, Mn(II), or Zn(II) [22], [26], [27]. These observations led to the identification of the relationship between extracellular Zn(II) and Mn(II) starvation in *S. pneumoniae*, and a concomitant increase in sensitivity to oxidative stress [26]. Although Mn(II) has many roles in cellular function [1], [28]–[30], it has a prominent contribution to oxidative stress management in many organisms [2]. Manganese serves in this capacity primarily as an essential cofactor of SOD [2], [31], but also by potentially substituting for ferrous iron in non-redox metabolic enzymes [32], and by providing direct protection against oxidative stress in organisms, such as *Neisseria gonorrhoeae* and *Lactobacillus plantarum*, that do not produce a Mn(II)-SOD [33], [34]. As a consequence, although our earlier observations were consistent with both the prior characterization of the pneumococcal SOD (SodA), which suggested that it functioned as a Mn(II)-cofactor containing SOD [35], and findings from related streptococcal species [36], [37], these data could not provide an unequivocal molecular explanation for how Mn(II) availability influenced oxidative stress management. Further confounding the issue, are recent studies of SODs from several streptococcal species that have shown these enzymes to be cambialistic proteins that utilize mixed iron/manganese cofactors [36], [38]. Thus, SodA from *S. pneumoniae* may not have an explicit requirement for Mn(II), suggesting that the metal ion could also potentially contribute to oxidative stress management via other mechanisms. Hence, the role of Mn(II) in protection against oxidative stress in *S. pneumoniae* requires further elucidation.

In this study we further investigated the molecular basis for the requirement of Mn(II) to identify the mechanisms by which extracellular Zn(II) could exert a toxic effect on the pneumococcus. By use of *in vitro* and *in vivo* analyses of *S. pneumoniae* our findings reveal that Zn(II) induced Mn(II) starvation in *S. pneumoniae* in a competitive manner. This resulted in an increase in sensitivity to oxidative stress that occurred concomitantly with a decrease in *sodA* transcription. Although the majority of physiological protection from oxidative stress in the pneumococcus arose from SodA, the Mn(II) ion was also shown to provide a low level of protection. Collectively, these findings provide further insight into the molecular basis of Zn(II) toxicity to *S. pneumoniae*, whilst also being broadly applicable to other bacteria that employ related pathways for Mn(II) acquisition.

## Results

### Extracellular zinc competes for manganese uptake by *S. pneumoniae*

**Figure 1:**
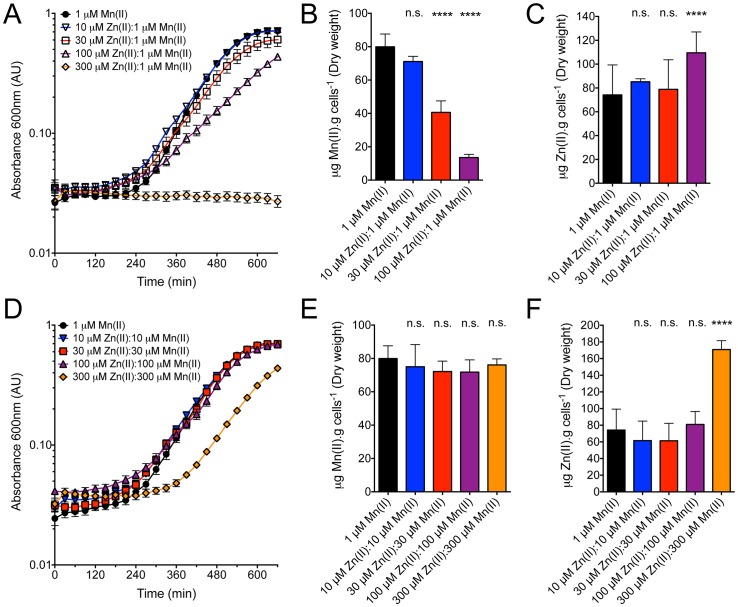
*In vitro S. pneumoniae* growth and metal ion accumulation. (**A**) Growth curves of *S. pneumoniae* grown in CDM with the following Zn(II):Mn(II) ratios (in μM): 300:1 (orange line, open diamond), 100:1 (purple, open triangle), 30:1 (red, open square), 10:1 (blue, open inverted triangle), and 1 µM Mn(II) (black, filled circle), respectively. Data are mean (± SEM) absorbance measurements from three independent biological experiments. Error bars, where not visible, are overlapped by the data points. (**B** and **C**) *S. pneumoniae* total cellular accumulation of Mn(II) (B) and Zn(II) (C) determined by ICP-MS of cells grown in following Zn(II):Mn(II) ratios (in μM): 100:1 (purple), 30:1 (red), 10:1 (blue), and 1 µM Mn(II) (black). Data are mean (± SEM) µg metal.g dry cell mass^−1^ from duplicate measurements of at least 3 independent biological experiments. (D) Growth curves of *S. pneumoniae* grown in CDM with the following Zn(II):Mn(II) ratios (in µM): 300:300 (orange line, filled diamond), 100:100 (purple, filled triangle), 30:1 (red, filled square), 10:1 (blue, filled inverted triangle), and 1 µM Mn(II) (black, filled circle), respectively. Data are means (± SEM) A_600_ measurements from three independent biological experiments. (**E** and **F**) *S. pneumoniae* total cellular accumulation of Mn(II) (E) and Zn(II) (F) determined by ICP-MS of cells grown in following Zn(II):Mn(II) ratios (in μM): 300:300 (orange), 100:100 (purple), 30:30 (red), 10:10 (blue), and CDM + 1 µM Mn(II) (black). Data are mean (± SEM) µg metal.g dry cell mass^−1^ from duplicate measurements of at least 3 independent biological experiments. Statistical significance of the differences in the means was determined by a two-tailed unpaired *t*-test (n.s. corresponds to not significant and **** to *P* value < 0.0001).

Results from our group and others have previously shown that Mn(II) homeostasis in *S. pneumoniae* can be influenced by Zn(II) concentrations and, when in large excess relative to Mn(II), result in a reduction in cell-associated Mn(II) [26], [39], [40]. However, in our prior studies the extent to which Zn(II) quantitatively perturbed Mn(II) acquisition was restricted to two concentrations [10 µM Zn(II):1 µM Mn(II) and 100 µM Zn(II):1 µM Mn(II)] [26]. In this study we sought to further analyze the effect of Zn(II) on *S. pneumoniae* D39 cell-associated Mn(II) when grown in a range of increasing Zn(II):Mn(II) ratios in CDM (Fig. 1A). Consistent with our previous observations, we observed that as the ratio of Zn(II) increased, relative to 1 µM Mn(II), *S. pneumoniae* showed a stepwise reduction in its growth rate. Inductively coupled plasma mass spectrometry (ICP-MS) analysis of cells grown in CDM supplemented with 1 µM Mn(II), had mean Mn(II) and Zn(II) accumulation values of 80±3 µg Mn(II).g cells^−1^ (*n* = 6) and 74±10 µg Zn(II).g cells^−1^ (*n* = 6), respectively (Fig. 1B,C). Consistent with our prior observations [26], the 10 µM Zn(II):1 µM Mn(II) show no significant change in either the growth rate or metal accumulation. Here we show, for the first time, that growth in the presence of 30 µM Zn(II):1 µM Mn(II) resulted in a minor, but significant, decrease in the growth rate (Fig. 1A) and resulted in a 2-fold reduction in Mn(II) accumulation (*P* < 0.0001) (Fig. 1B). Despite this, Zn(II) accumulation in *S. pneumoniae* was not observed to significantly increase (*P* = 0.73) (Fig. 1C). The 100 µM Zn(II):1 µM Mn(II) ratio induced a significant perturbation in the growth rate *S. pneumoniae* (Fig. 1A) and, consistent with our prior studies [26], [40], metal accumulation was also significantly affected, with Mn(II) accumulation reduced by 5.9-fold (*P* < 0.0001) (Fig. 1B), whilst Zn(II) accumulation showed a minor increase of 1.4-fold (*P* = 0.0091) (Fig. 1C). At ratios of 300 µM Zn(II):1 µM Mn(II) (Fig. 1A) or greater (data not shown) growth of *S. pneumoniae* was completely inhibited and thus precluded quantitative assessment of metal accumulation.

Previously we have observed that the phenotypic effect of Zn(II) on pneumococcal growth could essentially be ablated by supplementation with an equimolar concentration of Mn(II) [26]. In this study we observed a similar trend, with the sole exception being the 300 µM Zn(II):300 µM Mn(II) growth condition (Fig. 1D). Here, we observed that although supplementation with 300 µM Mn(II) could restore pneumococcal growth, at what was an otherwise inhibitory concentration [300 µM Zn(II):1 µM Mn(II); Fig. 1A], the growth rate still showed a significant delay and overall reduction by comparison to the unchallenged wild-type growth rate. This indicated that cellular processes other than Mn(II) uptake were being detrimentally affected during growth in the presence of very high levels of extracellular Zn(II). We then sought to ascertain whether supplementation with equimolar Mn(II), in the presence of Zn(II), had restored Mn(II) levels as the phenotypic growth experiments suggested. We show here, for the first time, that cell-associated Mn(II) concentrations were indeed restored to unchallenged levels when Mn(II) was supplemented at equimolar ratios to Zn(II) (Fig. 1E). Further, the concentrations of Zn(II) accumulated under these conditions were essentially the same as the non-challenged concentrations (Fig. 1F). The only exception was the 300 µM Zn(II):300 µM Mn(II) growth condition that showed a ∼2.5-fold (*P* < 0.0001) increase in Zn(II) (Fig. 1F).

**Figure 2:**
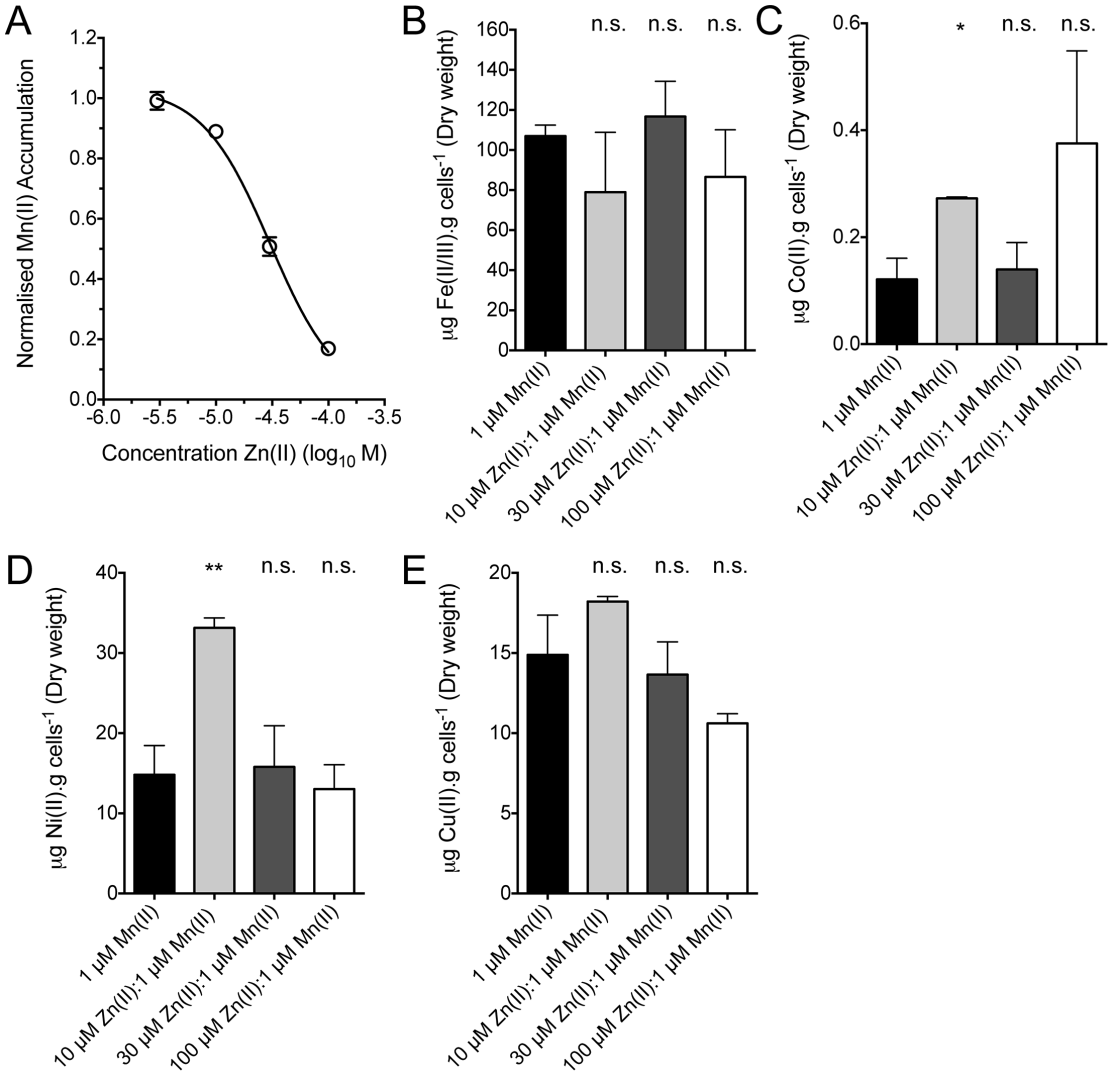
Competitive effect of Zn(II) on metal ion accumulation. (**A**) The concentration response curve fitting data for Mn(II) accumulation in *S. pneumoniae* D39 under extracellular Zn(II) stress. Data were normalized by comparison with non-competitive growth conditions [CDM + 1 µM Mn(II)]. Curve fitting was performed in Graphpad Prism version 5.0d (Graphpad). (**B**, **C**, **D**, and **E**) *S. pneumoniae* total cellular accumulation of Fe(II/III) (B), Co(II) (C), Ni(II) (D), and Cu(II) (E), determined by ICP-MS, when grown in CDM supplemented with 1 µM Mn(II), 10 µM Zn(II):1 µM Mn(II), 30 µM Zn(II):1 µM Mn(II), and 100 µM Zn:1 µM Mn. Data are mean (± SEM) µg metal.g dry cell mass^−1^ measurements from duplicate measurements of at least 3 independent biological experiments. The statistical significance of the differences in concentrations was determined by a two-tailed unpaired *t*-test (n.s. corresponds to not significant, * to *P* value < 0.05, and ** to *P* value < 0.01).

Analysis of the quantitative metal accumulation data for Mn(II) collected in this study revealed that Zn(II) had an EC_50_ for Mn(II) accumulation at a ratio of 30.2 µM Zn(II):1 µM Mn(II) in CDM (Fig. 2A). Therefore, the effect of extracellular Zn(II) on Mn(II) accumulation was consistent with competitive phenomena, which previously could only be inferred [26]. To assess whether extracellular Zn(II) affected the accumulation of other metal ions we further investigated the impact of competitive Zn(II) concentrations. As can be seen in Figures 2B-E, the effect of extracellular Zn(II) on metal accumulation was primarily restricted to Mn(II) accumulation, with no significant reduction in the accumulation of other transition row metals observed at any of the competitive ratios examined. However, Co(II) and Ni(II) did show minor increases at 10 µM Zn(II): 1 µM Mn(II), but as accumulation of these metal ions were not observed at other concentrations the significance of these changes were not readily apparent. Overall, these data indicate that the competitive effect of Zn(II) was primarily restricted to the Mn(II) uptake pathway. Collectively, these data provide direct quantitative evidence that extracellular Zn(II) competitively inhibits Mn(II) uptake in *S. pneumoniae* in a dose dependent manner and that the effect of Zn(II) primarily occurs on this pathway with negligible effects on other transition row metal ion transporter or the Zn(II) homeostatic mechanisms.

### Zinc-induced manganese starvation results in hypersensitivity to oxidative stress

Loss of Mn(II) uptake has previously been associated with a reduction in the ability of *S. pneumoniae* to survive chemically- and immune effector-cell mediated oxidative stress [23], [26], [41]. However, the molecular basis underlying this susceptibility has been unclear. Prior studies by our group and others have reported that a *S. pneumoniae* Δ*psaA* mutant strain is hypersensitive to both H_2_O_2_ and paraquat mediated oxidative stress [23], [26], [41]. Paraquat causes oxidative damage by promoting a futile redox cycle in the cytoplasm that generates superoxide radicals. In principle, these ROS would be detoxified by the pneumococcal superoxide dismutase (SodA). However, it has also been reported that supplementation of the Δ*psaA* strain with Mn(II) failed to restore protection from paraquat despite the mutant strain showing near wild-type levels of SOD activity [41]. Consequently, in this work we sought to further elucidate the connection between Mn(II) and resistance to oxidative stress. By manipulating Zn(II) concentrations in CDM it is possible to modulate the efficacy of Mn(II) uptake by *S. pneumoniae* and thereby delineate the effects of Mn(II) and SodA in pneumococcal response to ROS.

**Figure 3:**
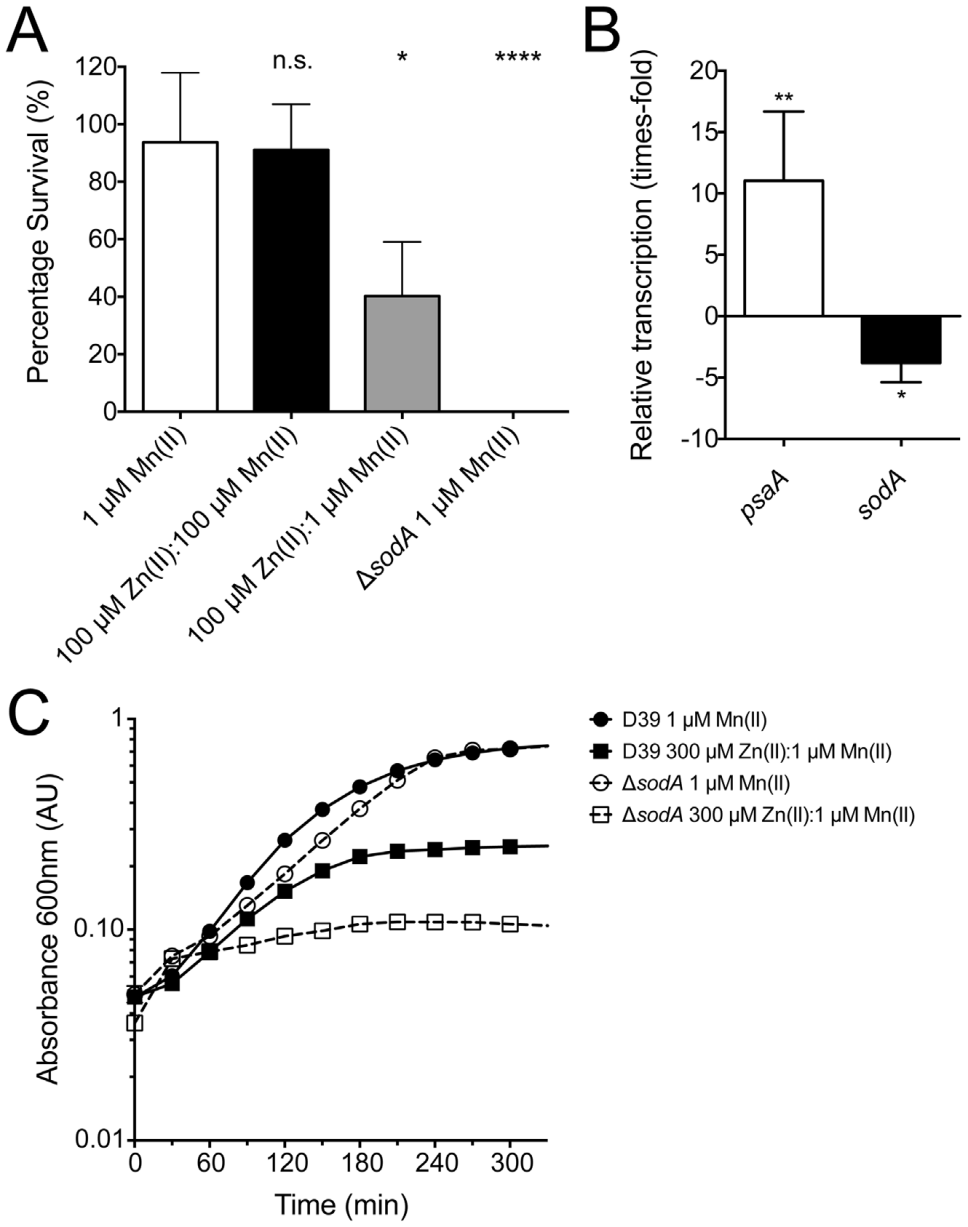
*S. pneumoniae* response to oxidative stress. (**A**) Paraquat killing of the *S. pneumoniae* wild-type (D39) and Δ*sodA* mutant grown in CDM + 1 µM Mn(II) (white), and *S. pneumoniae* (D39) grown in 100 µM Zn(II):100 µM Mn(II) (black) or 100 µM Zn(II):1 µM Mn(II) (light gray) conditions. Survival was calculated as a percentage of c.f.u. after 30 minutes paraquat challenge compared to 30 minutes without challenge. The experiment was performed with 3 independent biological samples and data are the means (± SEM). The statistical significance of the differences in mean survival was determined by a two-tailed unpaired *t*-test (n.s. corresponds to not significant, * corresponds to *P* value < 0.05, and **** P value < 0.0001). (**B**) *S. pneumoniae* D39 mRNA transcription levels were examined after growth in CDM + 1 µM Mn(II) or 100 µM Zn(II):1 µM Mn(II). Real-time RT-PCR data for the indicated conditions were normalized against those obtained for the 16S rRNA control. Data are means (± SEM) of at least three biological replicates. The statistical significance of the differences in relative transcription level was determined by a two-tailed unpaired *t*-test (* corresponds to *P* value < 0.05, and ** to *P* value < 0.01). (**C**) *S. pneumoniae* D39 (filled) and Δ*sodA* (open) were grown in CDM supplemented with 1 µM Mn(II) until an A_600_ of 0.3 was reached. Cells were washed in CDM and then inoculated to an A_600_ of 0.05 in CDM consisting of in CDM + 1 µM Mn(II) (circle) or 300 µM Zn(II):1 µM Mn(II) (square). Data are means (± SEM) A_600_ measurements from three independent biological experiments. Error bars, where not visible, are overlapped by the data points.

Previously we showed that during growth in 100 µM Zn(II):1 µM Mn(II) *S. pneumoniae* survival, when challenged with paraquat, was significantly reduced [26]. In this study we observed a similar effect with a significant reduction in survival to 32% (*P* = 0.0252), by comparison with growth in CDM with 1 µM Mn(II) (Fig. 3A). Here we show that upon supplementation with an equimolar ratio of Mn(II), wild-type resistance to paraquat exposure could be restored (Fig. 3A). Taken together these data indicate that resistance to paraquat exposure directly correlates with Mn(II) accumulation and was independent of the Zn(II) concentration in the extracellular medium. These observations are also consistent with the hypothesis that the pneumococcus utilizes Mn(II) as a cofactor for SodA, as has previously been implicated for *S. pneumoniae* and other streptococcal species [35], [36], [38]_ENREF_11. To further examine the effect of Zn(II)-induced Mn(II) starvation, we analyzed the effect of the 100 µM Zn(II):1 µM Mn(II) treatment on *S. pneumoniae* by qRT-PCR. The transcription of *sodA* was significantly down-regulated by 3.8-fold (*P* value  = 0.0078). This occurred concomitantly with a significant increase in *psaA* transcription of 11.3-fold (*P* value  = 0.0049) (Fig. 3B). The observed down-regulation of *sodA* transcription was similar to that previously reported for the *S. pneumoniae* D39 Δ*psaA* strain and this provided further support for the inference that the down-regulation of *sodA* was due to a Mn(II)-specific regulatory effect independent of Zn(II) concentrations [31]. Collectively, these data show that *sodA* transcription is regulated by Mn(II) abundance, and it is the resulting loss of Mn(II) that leads to a reduction in *sodA* transcription, which correlates with the heightened sensitivity to oxidative stress.

### Sensitivity of *S. pneumoniae* to oxidative killing is primarily dependent on SodA

We then constructed a mutant strain deficient in SodA to ascertain whether Mn(II) was capable of directly protecting against paraquat exposure or whether SodA was required. The mutant strain showed almost wild-type growth (Fig. 3C) and ICP-MS analysis confirmed that loss of the *sodA* gene had no effect on metal accumulation, with the mutant strain showing wild-type accumulation of Mn(II) (80±6 µg Mn(II).g cells^−1^ [*n* = 8]) and Zn(II) (72±6 µg Zn(II).g cells^−1^ [*n* = 8]). However, upon treatment with paraquat the Δ*sodA* strain demonstrated hypersensitivity to oxidative killing with less than 1% survival (Fig. 3A). Therefore, it can be concluded that SodA has a crucial role in protection against paraquat mediated oxidative stress.

To further investigate whether protection against oxidative stress during exponential growth required Mn(II) or SodA, Mn(II)-replete exponential phase wild-type *S. pneumoniae* were challenged with a concentration of Zn(II) [300 µM Zn(II):1 µM Mn(II)] that would prevent any subsequent Mn(II) uptake, leading to depletion of endogenous Mn(II) by cell division. Figure 3C shows that wild-type *S. pneumoniae* was able to grow for approximately 180 minutes before cell growth stopped in response to a high level of Zn(II) stress. By contrast, when exposed to 300 µM Zn(II):1 µM Mn(II) the Δ*sodA* strain stopped exponential growth within 60 minutes (Fig. 3C). Therefore, although the Δ*sodA* strain is hypersensitive to superoxide, it was the subsequent depletion of Mn(II) via extracellular Zn(II) that resulted in a more rapid attenuation in growth by comparison to the wild-type strain. Thus, it can be inferred that, although Mn(II) does provide some degree of protection against oxidative stress independently of SodA, protection against endogenous oxidative stress appears to predominantly arise from the action of SodA. Taken together, these results demonstrate that Mn(II) has a crucial role in *S. pneumoniae* growth where it provides protection from oxidative stress, primarily associated with SodA, but also through a lower efficiency secondary mechanism.

### *S. pneumoniae* SodA is a cambialistic superoxide dismutase that can utilize Mn(II) and/or Fe(II) cofactors, but which is transcriptionally regulated by Mn(II)

**Figure 4:**
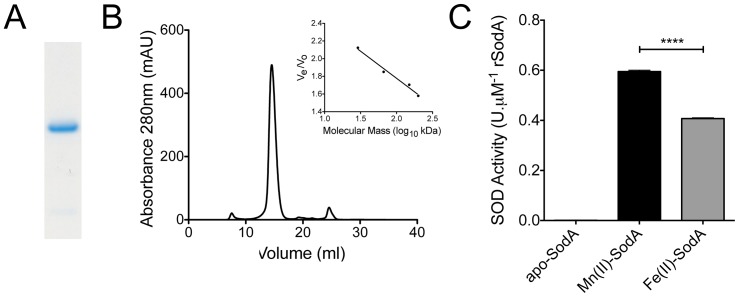
rSodA purification and characterization. (**A**) Purified rSodA electrophoretically separated on a 12.5% SDS polyacrylamide gel, with the major band stained by PAGE Blue. (**B**) Determination of the apparent molecular mass of the purified rSodA by gel-permeation chromatography on a Superdex 200 10/300 column. Inset is the linear regression of the protein molecular mass standards used to calibrate the column (carbonic anhydrase  = 29 kDa, bovine serum albumin  = 66 kDa, yeast alcohol dehydrogenase  =  150 kDa, sweet potato β-Amylase  =  200kDa). rSodA eluted with a calculated molecular mass of 60.8 kDa. (**C**) The SOD activity of apo-rSodA (white) and rSodA loaded with Mn(II) (black) or Fe(II) (light gray) was measured. The data are presented as SOD activity units (U) per µM protein. Data are means (± SEM) of three biological replicates.

Collectively, the above data indicate that *S. pneumoniae* resistance to oxidative stress depends upon Mn(II) accumulation to permit *sodA* transcription. In 2000 Yesilkaya and coworkers concluded that SodA contained a Mn(II)-cofactor on the basis of its susceptibility to chemical inhibitors [35]. However, we sought to obtain further insight into SodA by cloning and recombinantly expressing the *S. pneumoniae sodA* gene (rSodA). Purified rSodA, which had a molecular mass of ∼27.5 kDa under denaturing conditions (Fig. 4A), showed a native molecular mass of 60.8 kDa on gel permeation chromatography (Fig. 4B), consistent with the theoretical mass of a homodimer (∼55 kDa). Intriguingly, ICP-MS analysis revealed that the as-purified protein contained 0.17±0.0 mol Mn(II).mol monomer^−1^ and 0.72±0.0 mol Fe(II).mol monomer^−1^. As the acquisition of the metal cofactor by rSodA may have been influenced by the recombinant protein expression and culture medium used, the purified protein was subjected to denaturing chelation treatment and then reconstituted with either Mn(II) or Fe(II). The reconstituted isoforms of rSodA had metal:protein stoichiometries of 0.7±0.05 mol Mn(II).mol monomer^−1^ for the Mn(II) reconstituted protein and 1.0±0.04 mol Fe(II).mol monomer^−1^ for the Fe(II) reconstituted protein. Activity assays (Fig. 4C) showed that rSodA had the highest activity when loaded with Mn(II) (0.60±0.01 U. µM^−1^ rSodA_Mn_), but was active, albeit to a lesser extent, when loaded with Fe (0.40±0.01 U. µM^−1^ rSodA_Fe_). The apo-protein was essentially inactive (0.01±0.00 U. µM^−1^ rSodA) consistent with the requirement of a metal cofactor for function. Taken together, these data indicate that *S. pneumoniae* SodA is most likely a cambialistic enzyme capable of utilizing Mn(II) or Fe(II) cofactors similar to SodA from other streptococcal species [38].

**Figure 5:**
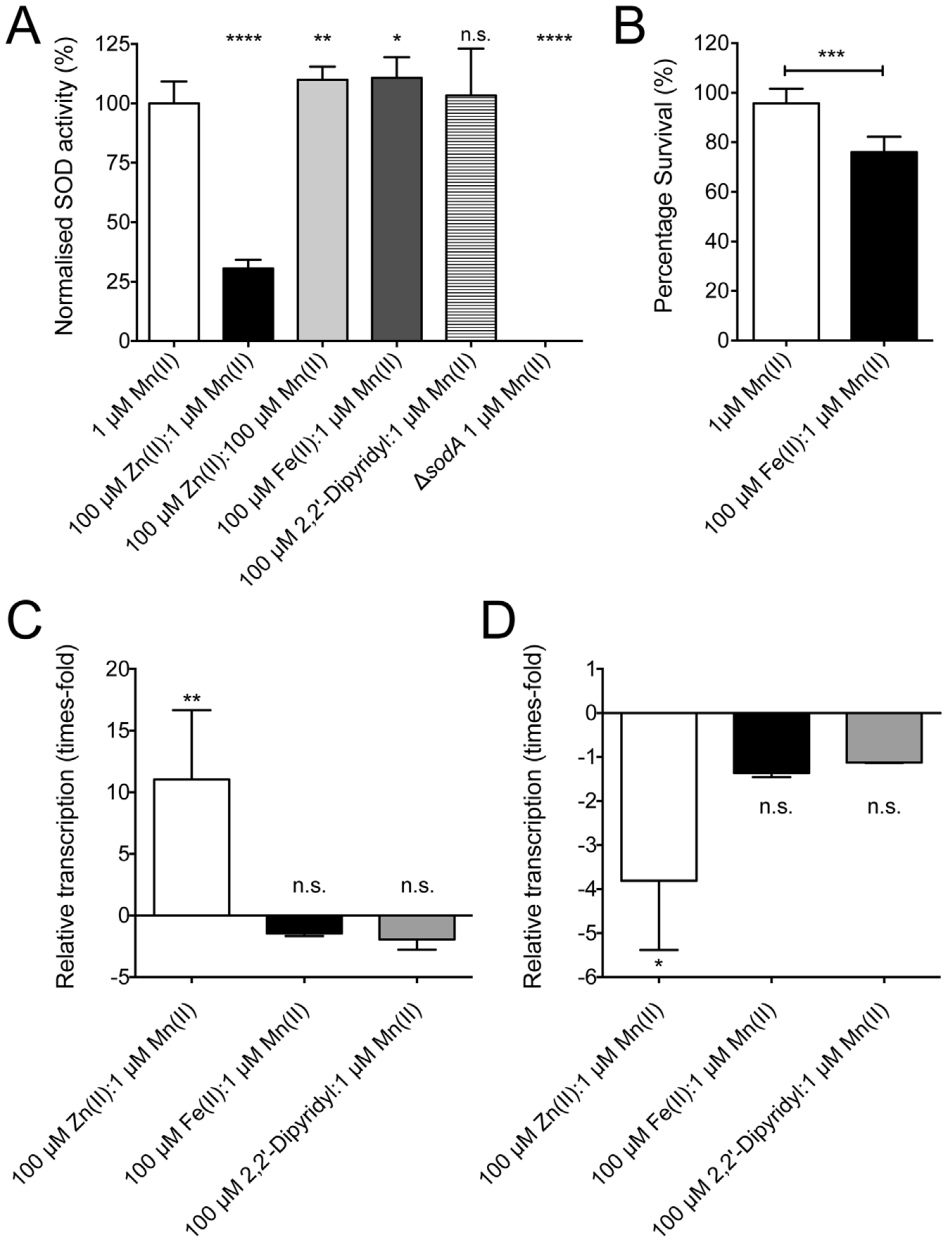
The effect of metal ions on SodA protection during oxidative stress. (**A**) Intracellular SOD activity of *S. pneumoniae* D39 grown in CDM supplemented with 1 µM Mn(II) (white), 100 µM Zn(II):1 µM Mn(II) (black), 100 µM Zn(II):100 µM Mn(II) (light gray), 100 µM Fe(II):1 µM Mn(II) (dark gray), 1 µM Mn(II) + 100 µM 2,2’-dipyridyl was measured (white striped). The *S. pneumoniae* Δ*sodA* mutant strain [1 µM Mn(II)] was included as a negative control. The activity units were corrected for total protein and normalized to *S. pneumoniae* D39 grown in CDM + 1 µM Mn(II). Data are means (± SEM) of duplicate reactions from three biological replicates. (**B**) Paraquat killing of *S. pneumoniae* D39 grown in CDM + 1 µM Mn(II) (white) or 100 µM Fe(II):1 µM Mn(II) (black) was assessed. Survival was calculated as a percentage of c.f.u. after 30 minutes paraquat challenge compared to 30 minutes without challenge. The experiment was performed with at least 3 independent biological samples and data are the means (± SEM). (**C** and **D**) *S. pneumoniae* D39 mRNA transcription levels of (C) *psaA* and (D) *sodA* were examined after growth in CDM + 1 µM Mn(II) (white), 100 µM Fe(II):1 µM Mn(II) (black) or 1 µM Mn(II) + 100 µM 2,2′-dipyridyl (light gray). Real-time RT-PCR data for the indicated conditions were normalized against those obtained for the 16S rRNA control. Data are means (± SEM) of at least three biological replicates. The statistical significance of the differences in observed means was determined by a two-tailed unpaired *t*-test (n.s. corresponds to not significant, * corresponds to *P* value < 0.05, ** to *P* value < 0.01, *** to *P* value < 0.001, **** to *P* value < 0.0001).

The cambialistic capacity of rSodA, similar to that reported for other streptococcal species [38], raised the possibility that its transcription may also be responsive to Fe(II). However, this seemed unlikely as an analysis of the upstream region of the *sodA* gene failed to reveal any known iron-transcriptional regulator sites. The effect of Fe(II) concentrations was assessed by examining the effect of *S. pneumoniae* grown in CDM supplemented with 1 µM Mn(II), high-Fe(II) [CDM supplemented with 100 µM Fe(II):1 µM Mn(II)], and low-Fe(II) [CDM with 1 µM Mn(II) treated with the specific Fe-chelating agent 2,2′-dipyridyl]. Analysis of cells grown in the presence of high-Fe(II) showed minor, but significant, increases in both Mn(II) (133±16 µg Mn(II).g cells^−1^ [*n* = 4]) and Zn(II) (148±11 µg Zn(II).g cells^−1^ [*n* = 6]) accumulation. Consequently, an excess of Fe(II) did not appear to negatively affect acquisition of other transition row metal ions. Analysis of whole cell extracts revealed that alterations in CDM Fe(II) concentrations did not affect superoxide dismutase activity despite increased sensitivity to oxidative stress seen under high-Fe(II) (Fig. 5A,B). Whereas the 100 µM Zn(II):1 µM Mn(II) treatment resulted in down-regulation of *sodA*, qRT-PCR analysis of gene expression of *psaA* and *sodA* between *S. pneumoniae* grown in high- or low-Fe relative to CDM + 1 µM Mn(II) suggested that if Fe(II) does regulate *sodA* transcription, it is subordinate to Mn(II) (Fig. 5C,D). Collectively, these data indicate that Mn(II) transcriptionally regulates sodA as modulation of Fe(II) concentrations in the media had no direct effect on its expression, despite the cambialistic nature of enzyme.

## Discussion

Recently we established that high concentrations of extracellular Zn(II) were associated with reduced Mn(II) accumulation by the pneumococcus due to impairment of the high-affinity Mn(II) permease, PsaBCA [26], [40]. In this study we have now provided quantitative data that directly shows that Zn(II) acts as a competitive inhibitor of Mn(II) uptake, supporting our earlier inference, and directly demonstrating that this occurs in a dose dependent manner. Analysis of other transition row metal ions revealed that the impact of Zn(II) on *S. pneumoniae* was restricted to Mn(II). This would be consistent with the pneumococcus only employing cluster A-I SBPs for the purpose of Zn(II)- or Mn(II)-recruitment. Intriguingly *S. pneumoniae* grown in 10 µM Zn(II):1 µM Mn(II) showed a minor but significant increase in Co(II) and Ni(II) accumulation. However, as an increase in the accumulation of these metals was not observed at any other competitive Zn(II) concentration the data does not indicate that this effect is directly related to increases in extracellular Zn(II). Cu(II) accumulation also fluctuated, but as the differences in concentrations never achieved significance, the data suggest that Cu(II) accumulation is less sensitive than Mn(II) to perturbation by extracellular Zn(II). Further support for the competitive model of Zn(II) inhibition of Mn(II) uptake can be derived from the observations that the phenotypic impact of extracellular Zn(II) on growth was abrogated, at almost all concentrations, by supplementation with Mn(II) and that cell-associated Mn(II) was similarly restored to unchallenged levels. This supports the inference that it is not the concentration of Zn(II) that is directly harmful to the pneumococcus but, instead, the ratio of Zn(II) to Mn(II) that results in the competitive inhibition of the Mn(II) ABC permease. Our data, and that interpretation, are also entirely consistent with the ∼70-fold greater affinity of PsaA for Mn(II) over Zn(II), which would predict that, in the presence of equimolar Mn(II) and Zn(II), PsaA would preferentially interact with Mn(II) [26]. However, this study also highlights the fact that the *in vitro* affinity constants do not reflect the cell-surface environment of the pneumococcus, as the observed EC_50_ for Zn(II) inhibition of Mn(II) uptake is greater than would be otherwise expected. Whether this arises from considerations of the Irving-Williams series with respect to protein-metal ion interaction [42], [43], the prevailing metal ion availability, or due to complexities of the protein and polysaccharide composition of the cell wall and membrane, requires further investigation.

This work also revealed an apparent limitation of PsaA. In almost all cases, extracellular Zn(II) had negligible effect on cell associated Zn(II). However, growth in 100 µM Zn(II):1 µM Mn(II) ratio led to a higher association of Zn(II) with total cellular material than might otherwise be expected if Zn(II) were not being specifically accumulated in the cytoplasm. Although the possibility that Zn(II) could be imported through PsaBCA cannot be completely excluded, recent *in vitro* characterization of PsaA showed that Zn(II)-binding to the protein was essentially irreversible [40]. Furthermore, this inference is also supported by a similar observation reported for the PsaA homolog, MntC, from *Staphylococcus aureus* [44]. Thus, we speculate that, concomitant with the up-regulation of *psaA* transcription due to Mn(II) depletion, the increase in Zn(II) at the cell-surface during growth in 100 µM Zn(II):1 µM Mn(II) arises from Zn(II) competing with Mn(II) and irreversibly binding to PsaA leading to Zn(II)-PsaA accumulating at the cell-surface.

At higher ratios of Zn(II), 300 µM Zn(II):300 µM Mn(II), even though growth could be restored, by contrast with the complete inhibition of growth at 300 µM Zn(II):1 µM Mn(II), it was not restored to unchallenged wild-type levels. This observation correlated with the increased association of Zn(II) with cellular material, even though the Mn(II) accumulation was restored to unchallenged levels. This could be explained by the higher ratio of extracellular Zn(II) to other cations facilitating its accumulation at the cell surface or leading to it being transported into the cell via pathway(s) independent of PsaA. Collectively, our data support a model whereby extracellular Zn(II) primarily competes for the SBP of the Mn(II) ABC permease. However, this work also shows that at very high ratios of Zn(II), independent of Mn(II) uptake, other transport processes are also susceptible to Zn(II) competition leading to deleterious cellular consequences, such as impaired biogenesis of iron sulfur cluster containing enzymes [45].

These observations are also significant in terms of host-pathogen interplay. In recent years, the ability of the host to exploit the essential nature of transition metals by restricting their availability has become increasingly understood and is now referred to as “nutritional immunity” [46], [47]. Although host restriction of iron has been studied for many decades [48]–[50], more recent work has identified that the bioavailability of Mn(II) and Zn(II) can also be modulated by the host organism during infection [26], [51], [52]. The host factors that facilitate these processes are still under investigation, but recent studies of the Gram-positive human pathogen *Staphylococcus aureus* have elegantly revealed that the host S100 protein calprotectin has a major role in cation sequestration and impedes staphylococcal infection [51], [53]. Our earlier finding, that Zn(II) abundance increased during pneumococcal infection, suggested that Zn(II) may also act as a component of nutritional immunity [26]. This supposition would correlate with the observations that the pneumococcal Mn(II) ABC permease is essential for infection [23], that pneumococcal burden increases in Zn(II)-deficient animal models [54], [55], and that dietary Zn(II) deficiency is one of the major factors associated with the incidence and severity of pneumococcal infections in children [56], [57]. Thus, the observations in this work offer a potential mechanism by which Zn(II) could act in nutritional immunity. Inhibition of the pneumococcal Mn(II) ABC permease occurred by a competitive mechanism wherein, as the ratio of Zn(II) relative to Mn(II) increased, the capacity of the ABC permease to import Mn(II) was decreased. Pneumococcal infection has previously been shown to result in an increase in Zn(II) relative to Mn(II) in the niches colonized by the pathogen, *i.e.* brain, lung, nasopharynx, and blood serum [26]. The Zn(II):Mn(II) ratios in all niches studied in that work would all exceed the EC_50_ [30 Zn(II):1 Mn(II)] observed for inhibition of Mn(II) uptake determined here. Taken together, these findings would be consistent with Zn(II) abundance serving to ablate Mn(II) uptake by a competitive mechanism. However, we would stress that we do not yet have direct evidence that Zn(II) is acting to inhibit pneumococcal colonization, as Zn(II) has many roles in immune function, and that further studies are required.

The heightened sensitivity of the pneumococcus to chemically induced oxidative stress when starved of Mn(II), is consistent with our prior observations and those in other streptococcal species [26], [36]–[38]. Here, we have directly shown the relationship between Mn(II) and SodA. It should be noted that *S. pneumoniae* was originally reported to contain two SODs, with SodA demonstrated to be the major SOD at a functional level and a weakly expressed secondary pneumococcal SOD suggested to be a Fe-SOD [35]. However, the identity of this secondary SOD remains unclear, as no other SOD-like genes are present in the *S. pneumoniae* genome and no similar reports of a second Fe-SOD have been reported in other streptococcal species. In this study we have focused exclusively on SodA, and the increased sensitivity to O_2_^−^ ions associated with Mn(II) starvation can be directly attributed to decreased *sodA* transcription. Despite our observations of manganese responsive regulation of *sodA*, PsaR, the regulator of the psa permease, did not regulate the gene. No consensus PsaR binding sites were identified in the vicinity of the *sodA* gene, consistent with recent studies of *psaR* deletion strains [58]. Furthermore, no regulatory motifs corresponding to other known regulatory proteins could be found in the upstream region of the *sodA* gene. Taken together, the absence of PsaR binding sites and the lack of a direct response of *sodA* to the other divalent cations, *i.e.* Zn(II) or Fe(II) supplementation in the media, suggests that an unknown Mn(II)-responsive regulator principally regulates *sodA*. Despite this, the *in vivo* physiological cofactor of SodA from the pneumococcus remains unclear. Recombinant SodA was found to have cambialistic SOD capability as evidenced by its ability to function with either Fe(II) or Mn(II) cofactors. If this does reflect the *in vivo* scenario, this may be beneficial for *S. pneumoniae* as cambialistic SODs have been shown to be more resistant to H_2_O_2_ inactivation than Fe-SODs [38]. However, we have no direct evidence that the observed *in vitro* cambialistic capacity of recombinant SodA also occurs in *S. pneumoniae* under physiological conditions. Despite this, recent studies from other streptococci have suggested that cambialistic SODs may be more common than anticipated and are not easily deduced from amino acid sequence analyses [36], [38].

An additional benefit of a cambialistic SOD for *S. pneumoniae* could arise from the lack of a known iron efflux pathway in the pneumococcus. As a consequence, SodA may also serve a role in Fe-homeostasis with Mn(II) and Fe(II) being able to exchange on the protein. Further investigation into the interplay between Fe(II) and Mn(II) homeostasis will be required to elucidate these aspects of SodA function. However, it cannot be discounted that there could be a cost associated with variations in ratio of Fe(II) and Mn(II) in the SodA metal-cofactor. Our observations showed that the activity of SodA varied depending on the metal cofactor. In a physiological context, changes in the metal ratios of SodA could result in alterations in the resistance profile of *S. pneumoniae* to oxidative stress, independent of variations arising from changes in *sodA* transcriptional levels. This model offers a potential explanation for the minor, but significant, reduction in *S. pneumoniae* cell survival that was observed for cells grown in the presence of high iron (Fig. 5B), as the Fe(II)-cofactor SodA showed a lower level of *in vitro* activity by comparison with the Mn(II)-cofactor containing isoform (Fig. 4C). Furthermore, the potential for modulation of the metal cofactor of SodA by virtue of metal abundance could have ramifications for growth in the presence of high extracellular Zn(II). Although Zn(II) did not appear to be directly responsible for the observed sensitivity to oxidative stress, as the sensitive phenotypes could be reversed by supplementation with Mn(II), we cannot exclude the possibility that Zn(II) could also be contributing to the phenotype. Despite the lack of direct redox activity, Zn(II) could, via mismetallation and inactivation of proteins such as SodA, perturb the intracellular redox balance of *S. pneumoniae* independent of any impact on Mn(II) uptake. However, although the potential contribution of Zn(II)-mismetallation cannot be discounted, overall our data supports the less speculative conclusion that the principal effect of extracellular Zn(II) competition is Mn(II) starvation, which results in decreased *sodA* transcription and a concomitant increase in sensitivity to oxidative stress.

Analysis of the Δ*sodA* strain confirmed that despite being replete for Mn(II), it was hypersensitive to chemically induced oxidative stress. However, the Mn(II) ion was able to provide near wild-type levels of protection from endogenous oxidative stress as abrogating Mn(II) uptake and allowing it to be depleted by cell-division led to a rapid attenuation of growth of the Δ*sodA* strain relative to the wild-type strain. Taken together these data indicate that the Mn(II) ion can directly protect against oxidative stress. Several models for how Mn(II) can directly provide protection against oxidative stress have recently been proposed, but the precise nature of this secondary mechanism in *S. pneumoniae* remains unclear. Irrespective of the mechanism, it is clear that SodA is much more efficient in providing protection against oxidative stress.

In conclusion, this study shows that extracellular Zn(II) depletes Mn(II) accumulation in *S. pneumoniae* via competitive inhibition of the PsaBCA permease. The Zn(II)-induced Mn(II)-starvation results in a loss of SodA and Mn(II) and thereby leads to a hypersensitivity towards oxidative stress. Collectively these findings are significant in the context of host nutritional immunity, as the ratios of Zn(II) to Mn(II) in those niches colonized by the pneumococcus [26] have previously been reported to be greater than the EC_50_ reported here. Thus, this work offers a potential mechanism by which Zn(II) could act as a component of nutritional immunity.

## Materials and Methods

### *In vitro* growth measurements

Frozen stock *S. pneumoniae* D39 and mutant isoforms were prepared as described previously [26]. The medium used for *in vitro* growth measurements was cation-defined C+Y medium (CDM) as specified previously [26], [59]. ICP-MS of CDM was routinely performed to determine metal ion concentrations of the unsupplemented media. For *in vitro* growth experiments, culture was added to CDM supplemented with 1 µM MnSO_4_ and then supplemented with additional MnSO_4_ and/or ZnSO_4_ to the provide the ratios of metal ions as specified. The presence of other transition row metal ion concentrations in the CDM, as indicated by ICP-MS, were 4–5 µM Fe(II/III), 100–200 nM Co(II), 100–200 nM Ni(II), 100–200 nM Cu(II). The starting A_600_ was 0.05 for all cultures. For the extracellular Zn(II) stress experiment a stock culture was added to CDM with 1 µM MnSO_4_ to a starting absorbance at 600 nm (A_600_) of 0.05 and grown to an A_600_ of 0.5. Cells were washed with CDM, pre-warmed to 37°C, and then reinoculated into CDM with 1 µM MnSO_4_ and supplemented with either 0 or 300 µM ZnSO_4_, to an A_600_ of 0.3. Cell growth was then monitored at A_600_. All analyses were carried out in at least biological triplicate. For ICP-MS analyses, cells were grown in 50 mL of CDM supplemented with metals as indicated. Cell growth was monitored to an A_600_ of 0.3 after which the cells were harvested by centrifugation at 3,750 x *g* for 15 minutes at 8°C and washed 3 times, at 3,750 x *g* for 15 minutes at 8°C, in PBS + 5 mM EDTA and then washed 3 times with PBS at 3,750 x *g* for 15 minutes at 8°C. The concentration of the chelating agent used had been verified to be in a sufficiently large molar excess (> 500-fold) relative to media metal ion concentrations to remove metal ions associated with the cell surface of *S. pneumoniae*. Cells were transferred to pre-weighed tubes and heated at 80°C overnight. The dry cell mass was determined and the material boiled at 95°C for 20 minutes in 35% HNO_3_. The metal-ion containing supernatant was collected by centrifugation at 14,000 x *g* for 30 minutes and diluted to a final concentration of 3.5% HNO_3_ for metal ion composition determination on an Agilent 7500cx ICP-MS (Adelaide Microscopy, University of Adelaide).

### Construction of the *S. pneumoniae* Δ*sodA* strain

Primers were designed to replace the *sodA* genes with a spectinomycin acetyltransferase gene, by overlap extension PCR [60]. Then, the *sodA* overhanging fragment was transformed into *S. pneumoniae* [59], [61]. The Δ*sodA* mutant was confirmed by DNA sequencing (Australian Equine Genetics Research Centre, The University of Queensland). Primer sequences are presented in Table S1.

### Expression and purification of rSodA

Recombinant SodA was generated by PCR amplification of *S. pneumoniae* D39 *sodA*, using primers listed in Table S1, and ligation independent cloning was used to insert the gene into a C-terminal dodecahistidine tag containing vector, pCAMcLIC01, to generate pCAMcLIC01-SodA. High level protein expression was performed in *E. coli* LEMO21(DE3) grown in an autoinducing TB medium (Overnight Express, Merck) for 18 hours at 30°C. Cells were harvested and disrupted at 30 kPSI by a Constant Systems cell disruptor and the soluble supernatant isolated by centrifugation at 4°C for 60 minutes at 120,000 x *g*. rSodA was isolated in a HisTrap HP column on an AKTA Purifier and was further purified on a Superdex 200 10/300 gel permeation column.

### Apo-rSodA generation, refolding, and ICP-MS

Demetallated (apo) rSodA was prepared by dialyzing the protein (10 ml) in a 20kDa MWCO membrane (Pierce) against 4 L of sodium acetate buffer, pH 3.7, with 20 mM EDTA. The sample was then dialyzed against 4 L of 20 mM Tris-HCl, pH 7.2, 100 mM NaCl, at 4°C. The sample was then recovered and centrifuged at 18,000 x *g* for 10 minutes to remove any insoluble material. The sample was analyzed for metal content by boiling 10 µM protein at 95°C for 30 minutes in 3.5% HNO_3_. Samples were analyzed on an Agilent 7500cx ICP-MS (Adelaide Microscopy, University of Adelaide). Apo-rSodA was reconstituted in the presence of 10-fold excess of MnSO_4_ or FeCl_2_ for 30 minutes on ice. The protein was desalted on a PD10 column (GE Healthcare) and then used in SOD assays (Sigma-Aldrich).

### Real-time RT-PCR

*S. pneumoniae* D39 for transcription analyses were cultured as described above. Cells were harvested by centrifugation at 3,750 x *g* for 15 minutes at 8°C and subsequently lysed using TRIzol (Invitrogen) and chloroform. Following phase separation by centrifugation, RNA was purified using an RNA isolation kit (Ambion) and treated with DNase I (NEB). The RNA was reverse transcribed using random hexamers and qPCR was performed using a Roche LC480 Real-Time Cycler, as described previously [59]. The primers are listed in Table S1 and were used at a final concentration of 200 nM per reaction. 16S rRNA was employed as a control. Amplification data were analyzed using the comparative critical threshold (2^−▵▵C^_T_) method.

### Bacterial killing assays

Bacteria were grown to an A_600_ = 0.3 in minimal media with or without Zn(II) supplementation, washed 3 times with PBS + 2.5 mM EDTA to remove excess cations and then 3 times with PBS. Cells were incubated for 30 minutes with 60 mM paraquat (Sigma-Aldrich) and then serially diluted and plated on blood-agar. Plates were incubated overnight at 37°C + 5% CO_2_. Survival was calculated as the percentage of colony forming units (c.f.u.) after 30 minutes of paraquat challenged compared to the number of c.f.u. after 30 minutes without paraquat challenge [26].

## Supporting Information

Table S1 **Oligonucleotide primers used in this study.**
(DOCX)
