# Supplementary material for: Extracellular Zinc Competitively Inhibits Manganese Uptake and Compromises Oxidative Stress Management in Streptococcus pneumoniae
Source: PLoS One. 2014 Feb 18;9(2):e89427. doi: 10.1371/journal.pone.0089427 (PMC3928430; doi:10.1371/journal.pone.0089427)
Supplement: Table S1 — Oligonucleotide primers used in this study. (DOCX) [file pone.0089427.s001.docx]

**Table S1. Oligonucleotide primers used in this study.**

| **Primer** | **Sequence (5’→3’)** |
| --- | --- |
| SodALIC1F | TGGGTGGTGGATTTCCTGCTATTATCTTACCAGAACTTCCA |
| SodALIC1R | TTGGAAGTATAAATTTCCTTTAGCAGCTGCGTACAA |
| SodA_del_1F | GGATGTGGAACTGGAGTTGG |
| SodA_del_1R | TTGTTCATGTAATCACTCCTTCGCCATCTGTAATACCTCTTTTTCTTT |
| SodA_del_2F | CGGGAGGAAATAATTCTATGAGTGATAGTTGGAGGGAAGAATTG |
| SodA_del_2R | ACAGTCGACCTGAGTGGTCA |
| qD39_16S_F | CATGCAAGTAGAACGCTGAA |
| qD39_16S_R | TGTCATGCAACATCCACTCT |
| qPsaA1F | AGCCTATGGTGTTCCAAGTG |
| qPsaA1R | GTTTTCATTGGACGGTCATC |
| qSodA1F | ACACTTGAACCACGCTCTTT |
| qSodA1R | CAACCTGAACCAAAACGAGT |
